# Pathogenesis and Host Responses to Viral Diseases in Livestock Species

**DOI:** 10.3390/v15040925

**Published:** 2023-04-06

**Authors:** Fernando Vicosa Bauermann, Mayara F. Maggioli

**Affiliations:** Department of Veterinary Pathobiology, College of Veterinary Medicine, Oklahoma State University, Stillwater, OK 74078, USA

Infectious diseases in livestock species are responsible for significant economic losses worldwide and constantly threaten food security. Understanding viral pathogenesis and host immune responses elicited by viral pathogens is critical for efficient disease control. The Special Issue “Pathogenesis and Host Responses to Viral Diseases in Livestock Species” has published eight articles, including reviews and research papers. The diversity in research fields and worldwide experts contributing to this Special Issue is remarkable.

An exciting study by Giannitti et al. has demonstrated the bovine polyomavirus-1 (BoPyV-1) pathogenicity in cattle [1]. BoPyV-1 was identified in an aborted bovine fetus and caused severe tubulointerstitial nephritis, necrosis in tubular epithelial cells, tubular and interstitial inflammation, and interstitial fibroplasia [1]. In addition, abundant intranuclear viral inclusions and BoPyV-1 large T (LT) antigen were found in renal tubular epithelial cells [1].

Three papers were related to viruses considered part of the bovine respiratory disease complex (BRDC), and two of those investigated pestiviruses [2,3]. HoBi-like pestiviruses (HoBiPeV), a recently identified pestivirus in cattle and closely related to bovine viral diarrhea virus (BVDV), was demonstrated to be highly prevalent in cattle herds in Northern Brazil (Amazon region) [3]. The seropositivity to HoBiPeV (20.9%) was comparable to BVDV-1 (19.8%) [3]. Another pestivirus study evaluated the effect of the combined use of BVDV-modified live vaccine (MLV) and killed vaccines (KV) in heifers [2]. Notably, the frequency of IFN-mRNA positive CD4+, CD8+, and CD335+ populations was increased in animals administered MLV prior to KV compared to KV followed by MLV [2]. These results are critical for optimizing vaccine-induced protective responses against pestiviruses in cattle. Another member of the BRDC, the bovine herpesvirus 1 (BHV-1), was the topic of a review focusing on the molecular mechanisms of BHV-1 latency-reactivation in cattle [4]. The comprehensive review focuses on the latest findings on latency-reactivation by stress and reproductive hormones, including the activation of the β-catenin-dependent Wnt pathway by the glucocorticoid receptor signaling and other cooperative pathways involving type 1 nuclear androgen and progesterone receptors and the stress-induced Krüppel-like transcription factors KLF4 and KLF15 [4].

Another interesting review article focused on the advancements in the diagnosis, control, and clinical presentation of lumpy skin disease (LSD) in cattle and buffalos. LSD is a rising concern due to its recent spread through Asian countries, causing significant outbreaks in the region [5]. Senecavirus A (SVA), a swine pathogen, was also shown to infect bovine cell lines and peripheral blood mononuclear cells from cattle. However, the experimental infection of calves with SVA was unsuccessful [6], indicating that further research is needed to understand the susceptibility and potential impact of SVA on cattle.

High-consequence animal diseases were the subject of two research articles. Epitopes were mapped in the highly immunogenetic African swine fever virus (ASFV) CP312 protein, and findings may support the improved diagnosis and control of ASFV [7]. Advancements in the pathogenesis of foot-and-mouth disease virus (FMDV) were also described with the characterization of the protease activity of FMDV 3C^pro^ on the degradation of the connective-tissue BP180 protein and blister formation [8].

This Special Issue covers a wide range of veterinary-relevant virus research that undoubtedly contributes to a deeper understanding of infectious diseases in livestock species. This information is valuable for addressing the challenges posed by viral diseases and advancing their diagnosis and control.

## References

[1] Giannitti F., da Silva Silveira C., Bullock H., Berón M., Fernández-Ciganda S., Benítez-Galeano M.J., Rodríguez-Osorio N., Silva-Flannery L., Perdomo Y., Cabrera A. (2022). Bovine Polyomavirus-1 (*Epsilonpolyomavirus bovis*): An Emerging Fetal Pathogen of Cattle That Causes Renal Lesions Resembling Polyomavirus-Associated Nephropathy of Humans. Viruses.

[2] Falkenberg S.M., Dassanayake R.P., Crawford L., Sarlo Davila K., Boggiatto P. (2023). Response to Bovine Viral Diarrhea Virus in Heifers Vaccinated with a Combination of Multivalent Modified Live and Inactivated Viral Vaccines. Viruses.

[3] Baumbach L.F., Mósena A.C.S., Alves R.S., Camargo L.J., Olegário J.C., Lobraico L.R., Costa J.M.N., Borba M.R., Bauermann F.V., Weber M.N. (2023). HoBi-like Pestivirus Is Highly Prevalent in Cattle Herds in the Amazon Region (Northern Brazil). Viruses.

[4] Ostler J.B., Jones C. (2023). The Bovine Herpesvirus 1 Latency-Reactivation Cycle, a Chronic Problem in the Cattle Industry. Viruses.

[5] Datten B., Chaudhary A.A., Sharma S., Singh L., Rawat K.D., Ashraf M.S., Alneghery L.M., Aladwani M.O., Rudayni H.A., Dayal D. (2023). An Extensive Examination of the Warning Signs, Symptoms, Diagnosis, Available Therapies, and Prognosis for Lumpy Skin Disease. Viruses.

[6] Buckley A., Crawford L., Hoffman K., Falkenberg S. (2022). Experimental Senecavirus A Infection of Bovine Cell Lines and Colostrum-Deprived Calves. Viruses.

[7] Hagoss Y.T., Shen D., Zhang Z., Li F., Bu Z., Zhao D. (2023). Novel Epitopes Mapping of African Swine Fever Virus CP312R Protein Using Monoclonal Antibodies. Viruses.

[8] Ekanayaka P., Weerawardhana A., Chathuranga K., Park J.-H., Lee J.-S. (2022). Foot-and-Mouth Disease Virus 3Cpro Cleaves BP180 to Induce Blister Formation. Viruses.

